# Secular trends and customer characteristics of sweetened beverage and water purchasing at US convenience and other small food stores, 2014–2017

**DOI:** 10.1186/s12966-022-01268-2

**Published:** 2022-03-31

**Authors:** Megan R. Winkler, Kathleen Lenk, Darin Erickson, Melissa N. Laska

**Affiliations:** 1 Department of Behavioral, Social, and Health Education Sciences, Rollins School of Public Health, Emory University, 1518 Clifton Rd, Atlanta, GA 30322 USA; 2 Division of Epidemiology and Community Health, School of Public Health, University of Minnesota, Minneapolis, MN USA

**Keywords:** Sugar-sweetened beverages, Water purchasing, Corner stores, Convenience stores, Customer purchases, Trend analyses

## Abstract

**Background:**

Cardiovascular health is linked to sugar- and artificially-sweetened beverages (SSBs and ASBs). Prior studies document declines in SSB purchases. However, it is unclear if similar trends exist at convenience and other small food outlets, which often serve lower-income communities and where objective point-of-sales data are difficult to obtain. We examined trends (2014–2017) in observed SSB, ASB, and water purchases at convenience and other small stores as well as differences in purchasing by customer characteristics.

**Methods:**

We used observational purchase data collected annually (2014–2017) from 3010 adult customers at 147 randomly-sampled stores in Minneapolis/St. Paul, USA. SSB sub-types included any ready-to-drink sweetened soda, fruit, sport, energy, tea, or other drink, and ASBs included artificially-sweetened versions. Unsweetened water included ready-to-drink water. Mixed regression models examined trends over time and associations with customer characteristics, accounting for customers nested within stores and stores repeatedly measured over time.

**Results:**

Nearly 50% of purchases included an SSB. Approximately 10% included an ASB. There was no evidence of change over time in SSB or ASB purchasing. Customer purchasing of unsweetened water significantly increased over time (5.7 to 8.4%; *P* for trend = 0.05). SSB purchasing was highest among men, young adults, customers with lower education/ income, and customers that shopped frequently. ASB purchasing was highest among women, those 40–59 years, non-Hispanic White, Hispanic, and customers with higher education/ income.

**Conclusions:**

Despite research suggesting previous declines in SSB consumption and purchasing in the US, we identified a persistent, high trend of SSB purchasing overtime at convenience and other small food stores. Consumption of SSBs and water are growing targets for public policy and health campaigns. Results demonstrate additional work is needed curb sweetened beverage purchasing and promote water purchasing at convenience and other small food stores, which are often prevalent in low-income and marginalized communities.

## Introduction

Sugar-sweetened beverages (SSBs) are important contributors to obesity and cardiovascular health [1–7] and increasing evidence indicates similar concerning patterns with artificially-sweetened beverages (ASBs) [4, 5, 8, 9]. With this, decreasing consumption of SSBs and ASBs have become growing targets for public health and policy intervention [10–14]. Given the ongoing need for evidence-informed policy approaches, it remains important to understand sweetened beverage purchasing trends over time, characteristics of purchasers, and how interventions may need to be modified depending on the type of outlet where these beverages are purchased.

Recent US evidence using self-reported intake has documented declines in SSB consumption [15–19] and increases in both ASB [16] and water consumption [17, 20] across the past 10–20 years. Objective customer purchasing data are an important complement to self-reported intake data, avoiding self-report bias and other measurement challenges. Studies using purchasing data have documented similar declines in SSBs and increases in ASBs and water and relied on either point-of-sale data or household scanner data of Universal Product Codes [21–25]. One study examined purchasing across specific store types, such as pharmacies and supermarkets [21].

Convenience and other small food stores, such as corner stores, gas-marts, and dollar stores, are frequent venues for SSB purchasing [26–28], and research suggests these are key SSB purchasing outlets for low-income communities [29]. However, objective purchase data from convenience and other small food stores are often difficult to capture for two important reasons [30]. First, beverages at these venues are often purchased in small trips and immediately consumed, creating known data gaps in large consumer panel datasets (e.g., immediately consumable items, such as small drink bottles, have been identified as commonly missing from household scanner datasets) [31]. Second, many convenience and other small food stores lack point-of-sale systems due to high costs [30, 32]. Relying only on sales data from venues with point-of-sale technology introduces selection bias effects [29] and underrepresents purchasing in marginalized communities. Thus, it remains unclear if the observed declines in SSB consumption and purchasing from prior US studies reflect trends in purchases made in convenience and other small stores and the distribution of customers making these different purchases.

This study addresses these gaps by using objective (observed) purchasing data among adult customers at convenience and other small food stores in Minneapolis- St. Paul, USA. We examined trends (2014–2017) in the purchasing of SSBs, ASBs, and unsweetened water overall and by beverage sub-type (e.g., sugar-sweetened energy drinks, sugar-sweetened fruit drinks). We also examined whether purchasing varied across key customer characteristics relevant to understanding inequity (e.g., customers’ residential neighborhood socioeconomic status) and are commonly targeted by the beverage industry (e.g., age, race/ethnicity) [33–35]. Such work is important to support ongoing public health efforts to promote water, reduce sweetened beverage consumption, and understand how this work should be approached at convenience and other small food stores.

## Methods

### Study design

We used observational food and beverage purchasing data collected annually (2014–2017) as part of the STaple foods ORdinance Evaluation (STORE) study. The primary objective of the STORE study was to assess the effects of the Minneapolis Staple Foods Ordinance on the healthfulness of store environments and customer purchasing in small and non-traditional food stores [36]. The Minneapolis ordinance required vendors to stock a minimum amount and varieties of healthy staple foods (e.g., fruits, whole grains). However, SSBs, ASBs and water were not addressed in the policy. Data were collected in Minneapolis as well as in St. Paul, Minnesota, USA, which served as the comparison (i.e., control) site.

One-hundred and eighty eligible stores (90 Minneapolis, 90 St. Paul) were randomly selected based on administrative lists of licensed retailers. After a pre-data collection store visit, 23 stores were deemed ineligible and 2 stores did not provide consent, resulting in 155 stores that participated at one or more study time points. Additional details on store eligibility, including participation at each time point, have been previously published [36, 37]. All human subjects study protocols were approved by the University of Minnesota Institutional Review Board.

### Data collection and sample

To evaluate customer purchases, teams of two data collectors visited stores primarily on weekdays between 10 am and 7 pm to assess customer activity at the busiest times for stores*.* With manager permission, intercept interviews were performed with customers exiting the store. All customers that had a visible food, beverage, or bag of purchases and were not clearly under the age of 18 were invited to participate [38]. Customers who were at least 18 years old, had made a food or beverage purchase, and agreed to participate then participated in a direct observation of their purchased foods and beverages, and data collectors recorded details, including the product name, amount, and size. Data collectors also performed a brief interviewer-administered survey that collected information about customers’ socio-demographics, shopping behaviors, and self-reported height and weight. Across the four time points, a total of 3010 customers participated in intercept interviews at 147 stores, which reflected an overall customer response rate of 35%. Additional details on data collection methods, participant eligibility, and response rates are published elsewhere [36, 38].

Among the 147 stores (78 Minneapolis, 69 St. Paul) with customers participating at one or more time points, 40% were food-gas marts, 34% were convenience/corner stores, 15% were pharmacies, 10% were dollar stores, and < 1% were classified as a general retailer. Fifty-six percent of stores were corporate- or franchise-owned and 44% independently-owned. Nearly all (95%) stores were authorized to accept US Supplemental Nutrition Assistance Program customer benefits.

### Measures

#### SSB, ASB, and water purchases

Data on customer food and beverage purchases were entered by trained staff into the Nutrition Data System for Research, a software application that generates nutrition profiles for food and beverage product categories [39]. We defined a SSB, ASB, or unsweetened water purchase as any customer who purchased at least 4 fluid ounces of a ready-to-drink version (i.e., packaged beverage ready for consumption immediately after purchase). We examined the prevalence of customers who purchased these beverages overall and by sub-categories (see Table 1 for definitions).

**Table 1:** Category scheme to classify beverage purchases entered in the Nutrition Data System for Research into sugar-sweetened beverage (SSB), artificially sweetened beverage (ASB), and unsweetened water categories and sub-categories

Beverage category	Definition
**SSB**	
Regular Soda	Carbonated non-alcoholic beverage with added sugar or a combination of sugar and artificial sweeteners; sweetened soft drinks.
Fruit Drinks	Non-carbonated, non-alcoholic fruit drink with added sugar or a combination of sugar and artificial sweeteners; fruit drinks; does not include 100% fruit juices.
Sports Drinks	Sweetened sports drinks; hand-coded from nondairy-based sweetened meal replacement/ supplement beverage category; includes Gatorade, Powerade, and thirst quencher or sports type ready-to-drink beverages.
Energy Drinks	Sweetened energy drinks; hand-coded from nondairy-based sweetened meal replacement/ supplement beverage category; includes AMP Energy, Red Bull, Rockstar, Monster Energy, Venom, Full Throttle, and special formulated energy drink products.
Sweetened Tea	Sweetened tea drinks; tea with added sugar or a combination of sugar and artificial sweeteners.
Other SSBs	Ready-to-drink sweetened water; dairy/milk-based drinks with added sugar; sweetened coffee/ coffee substitute drinks; combined due to limited purchases across time points.
Overall	Any purchase from the SSB sub-categories above.
**ASB**	
Diet Soda	Carbonated non-alcoholic beverage with only artificial or non-nutritive sweeteners (no added sugar); artificially sweetened soft drinks.
Other ASBs	Ready-to-drink artificially sweetened fruit drinks; nondairy-based artificially sweetened meal replacement/ supplement drinks, including artificially sweetened sports and energy drinks; artificially sweetened tea; artificially sweetened water; artificially sweetened milk/dairy-based drinks; artificially sweetened coffee/ coffee substitute drinks; combined due to limited purchases across time points.
Overall	Any purchase from either ASB sub-category above.
**Water**	
Overall	Ready-to-drink unsweetened bottled water; includes unsweetened coconut water.

*ASB* artificially sweetened beverage, *SSB* sugar-sweetened beverage

#### Customer characteristics

We examined both individual and residential neighborhood characteristics of customers. Individual characteristics included: age group (18–39 years, 40–59 years, or ≥ 60 years); sex (female or male); race/ethnicity (categorized based on frequency distributions into non-Hispanic White, non-Hispanic Black, Hispanic, or other non-Hispanic race); education (high school diploma or less, some college, or Bachelor’s degree or higher); annual household income (<$25,000, $25,000–$50,000, or > $50,000); body mass index (BMI; derived from self-reported height and weight and categorized into < 25.0, 25.0–29.9, and ≥ 30.0); and shopping frequency at the recruited small food store (at least daily, 1–6 times per week, or less than once per week). Categories were informed by prior studies [15, 17] and cell size.

Customers’ residential neighborhood characteristics were derived from their residential census tract, identified based on customer reports of the street name they live on and the nearest cross street. We categorized customers’ residential census tracts by poverty status and food desert status using data from the 2013–2017 American Community Survey (ACS) [40] and the USDA Food Access Research Atlas [41], respectively. Following prior work [37, 42], we defined high poverty neighborhoods as census tracts with > 50% of residents below 185% of the federal poverty level [43] (FPL) and less poverty neighborhoods as census tracts with ≤50% of residents below 185% FPL. Food desert neighborhoods were defined as census tracts that were both low-access (100 or more households without access to a vehicle and located >½ mile from nearest supermarket) and low-income (either a poverty rate > 20% or the census tract’s median family income is ≤80% of the state or metropolitan area’s median family income) [41].

### Statistical analysis

We first calculated descriptive statistics for all measures, including customer characteristics and beverage purchases, for each study year. To examine trends over time (2014–2017) in customer SSB, ASB, and water purchasing, we began by identifying whether there were changes in customer participation across time. Both customer race/ethnicity and BMI varied across time and were subsequently treated as covariates. We then computed separate mixed regression models for each beverage type to examine the purchasing rate across time controlling for customer race/ethnicity and BMI, a fixed city effect for the study design (Minneapolis/ St. Paul), and a random effect for store to account for nesting of customers within stores, the variation in customer interviews conducted per store, and stores repeatedly measured over time. Results are presented as predicted probabilities and the *p*-value for the linear time trend test (df = 1). As a sensitivity check, we examined whether predicted change over time varied by city, given a SSB public health campaign (i.e., “reTHINK your drink!”) was initiated in Minneapolis during the study period [44]; we did not identify any notable interactions between city and time and do not report results.

We then examined differences in beverage purchases by customer characteristics in an additional set of mixed regression models. Models examined each customer characteristic separately controlling for time trends, confounders (customer age, race/ethnicity, and sex), a fixed city effect for the study design, and a random effect to account for nesting of customers within stores and unbalanced number of customer observations. Results are presented as predicted percentages of customers with standard errors.

All analyses were performed using SAS 9.4 (SAS Institute, Cary NC) with significance set at α ≤ 0.05.

## Results

The analytic sample included 3010 adult food and beverage customers of convenience and other small food stores in Minneapolis and St. Paul, MN, USA. Table 2 reports sample sizes and proportions of customer characteristics across time.

**Table 2:** Characteristics of customers (*n* = 3010) at convenience and other small food stores, Minneapolis-St. Paul, USA, 2014–2017

	2014	2015	2016	2017
	N (%)	N (%)	N (%)	N (%)
**Individual Characteristics**				
Age				
18–39 years	332 (52)	452 (60)	457 (58)	420 (53)
40–59 years	230 (36)	247 (33)	273 (34)	275 (35)
> = 60 years	75 (12)	56 (7)	62 (8)	94 (12)
Sex				
Female	275 (43)	337 (44)	341 (43)	329 (41)
Male	358 (57)	423 (56)	459 (57)	469 (59)
Race/ethnicity				
Non-Hispanic White	295 (46)	310 (41)	314 (40)	297 (38)
Non-Hispanic Black	230 (36)	278 (37)	303 (38)	309 (39)
Hispanic	21 (3)	48 (6)	53 (7)	29 (4)
Non-Hispanic Other Racial Group^a^	90 (14)	122 (16)	124 (16)	155 (20)
Education				
High school diploma or less	244 (38)	273 (36)	323 (40)	305 (38)
Some college	227 (36)	297 (39)	288 (36)	308 (39)
Bachelor’s degree or higher	166 (26)	190 (25)	188 (24)	183 (23)
Household income				
< $25,000	216 (38)	259 (37)	266 (36)	274 (38)
$25,000–$50,000	201 (35)	230 (33)	251 (34)	236 (33)
> $50,000	156 (27)	210 (30)	220 (30)	211 (29)
Body Mass Index				
< 25	235 (37)	275 (37)	275 (36)	232 (30)
25–29.9	197 (31)	254 (34)	228 (30)	280 (37)
> =30	210 (33)	215 (29)	263 (34)	255 (33)
Shopping frequency				
At least daily	187 (29)	224 (29)	237 (30)	262 (33)
1–6 times per week	280 (44)	345 (45)	349 (43)	328 (41)
Less than 1 time per week	171 (27)	195 (26)	217 (27)	207 (26)
**Residential Neighborhood Characteristics**				
Poverty Status				
High Poverty (> 50% of residents < 185% FPL)	163 (28)	171 (25)	170 (23)	153 (21)
Less Poverty (≤50% of residents < 185% FPL)	429 (72)	518 (75)	577 (77)	560 (79)
Food Desert Status				
Low-income/ low-access neighborhood	166 (28)	191 (28)	200 (27)	182 (26)
Not in low-income/ low-access neighborhood	426 (72)	498 (72)	547 (73)	531 (74)

*FPL*, federal poverty level

^**a**^ Non-Hispanic Other Racial Group included: *n* = 9 Non-Hispanic Pacific Islander/Native Hawaiian, *n* = 99 Non-Hispanic Asian, *n* = 123 Non-Hispanic American Indian/ Native Alaskan, *n* = 128 Non-Hispanic More than 1 racial group, *n* = 132 Non-Hispanic Racial Category not captured

### Trends in SSB, ASB, and water purchases (2014–2017)

Nearly 50% of all observed food and beverage purchases included an SSB with approximately 25% of all purchases including soda across all study years (Table 3). There was no evidence of change over time between 2014 to 2017 in the proportion of customers purchasing SSBs overall or across beverage sub-types (*P* for trend > .05). A similar pattern was observed for ASB purchasing over time (*P* for trend > .05 overall and by sub-type), and ASB purchases were less commonly purchased among customers (approximately 8–9% overall). Unsweetened water purchases were less common than overall ASBs and SSBs, however, prevalence of purchasing significantly increased from 2014 to 2017 (5.7 to 8.4%; *P* for trend = 0.05).

**Table 3:** Trends in beverage purchases overtime at convenience and other small food stores, Minneapolis-St. Paul, USA, 2014–2017

Outcome	Predicted % of Customers by Year				***P*** value for linear trend
	2014	2015	2016	2017	(df = 1)
**Sugar-sweetened beverages**					
OVERALL	47.9	47.4	47.4	46.6	0.59
Regular Soda	27.3	26.6	26.2	25.6	0.37
Fruit Drinks	8.7	8.1	7.5	6.6	0.09
Sports Drinks	4.8	4.7	4.8	4.7	0.96
Energy Drinks	3.9	4.1	4.3	4.4	0.40
Sweetened Tea	5.1	4.8	4.7	4.2	0.37
Other Sweetened Beverages ^a^	2.5	3.0	3.5	4.1	0.16
**Artificially sweetened beverages**					
OVERALL	8.1	8.3	8.3	8.7	0.42
Diet Soda	5.7	5.3	5.1	4.7	0.78
Other Artificially Sweetened Beverages ^b^	2.7	3.1	3.2	4.0	0.10
**Unsweetened Water**	**5.7**	**6.5**	**7.2**	**8.4**	**0.05**

Models included store identification as a random effect due to nesting of customers within stores and controlled for city effect (study design) and two covariates identified as varying across time (race/ethnicity and BMI). Boldface indicates statistically significant change over time (*P* ≤ 0.05)

^a^ Includes ready-to-drink sweetened water, dairy/milk-based, and/or coffee-based drinks; combined due to limited cell size

^b^ Includes ready-to-drink artificially sweetened: fruit drinks, sports drinks, energy drinks, tea, water, and dairy/milk- and/or coffee-based drinks; combined due to limited cell size

### Differences in SSB, ASB, and water purchases by customer characteristics

Table 4 reports the differences in the prevalence of SSB, ASB, and water purchasing across customer characteristics. Compared to customers aged 18–39, those aged 40–59 and ≥ 60 years demonstrated significantly less SSB purchasing overall (52.5% vs 44.2 and 25.9%, respectively; *P* < .0001) as well as across fruit drink, sports drink, and energy drink SSB sub-categories. Customers aged 40–59 years had significantly higher purchasing than those aged 18–39 of overall ASBs (10.2% vs. 6.4%; *P =* 0.0006) and diet soda. Compared to men, women had significantly less purchasing of SSBs overall (37.6% v. 43.0%; *P =* 0.008) and energy drinks while significantly greater purchasing of overall ASBs (8.9% v. 6.0%; *P =* 0.002) and diet soda. Across racial/ethnic groups, overall SSB purchasing did not vary significantly, but purchasing by sub-type did. Compared to non-Hispanic White, non-Hispanic Black customers demonstrated significantly less purchasing of sports and energy drinks and significantly more purchasing of soda and fruit drinks. Compared to non-Hispanic White customers, non-Hispanic Black and other non-Hispanic racial groups displayed significantly less purchasing of ASBs overall (13.4% v. 2.6 and 6.1%, respectively; *P <* .0001) and diet soda, while there were no significant differences between Hispanic and non-Hispanic White customers.

**Table 4:** Prevalence of beverage purchases across customer characteristics (*n* = 3010 customers), Minneapolis-St. Paul, USA, 2014–2017

	SSBs						ASBs		Water^**a**^
	OVERALL	Soda	Fruit	Sports	Energy	Tea	OVERALL	Diet Soda	OVERALL
**Customer Characteristic**	Predicted % (SE)								
Age									
18–39 years (ref)	52.5 (1.8)	26.7 (1.6)	10.0 (1.0)	6.3 (0.7)	4.7 (0.7)	5.5 (0.7)	6.4 (0.8)	2.9 (0.5)	6.9 (0.8)
40–59 years	**44.2 (2.1)**	27.1 (1.7)	**6.0 (0.9)**	**2.7 (0.5)**	**2.2 (0.5)**	4.7 (0.8)	**10.2 (1.2)**	**7.3 (1.1)**	7.1 (0.9)
> = 60 years	**25.9 (2.6)**	**17.3 (2.3)**	**1.3 (0.7)**	**0.9 (0.7)**	**0.5 (0.3)**	2.6 (1.2)	5.9 (1.4)	4.9 (1.3)	8.2 (1.8)
Sex									
Male (ref)	43.0 (1.9)	24.1 (1.6)	4.0 (0.9)	2.6 (0.7)	2.3 (0.6)	4.9 (0.9)	6.0 (0.8)	3.5 (0.7)	7.4 (0.9)
Female	**37.6 (2.2)**	22.6 (1.9)	4.5 (1.0)	2.4 (0.7)	**1.3 (0.4)**	3.4 (0.7)	**8.9 (1.2)**	**6.4 (1.0)**	7.4 (0.9)
Race/ethnicity									
Non-Hispanic White (ref)	40.1 (2.0)	21.7 (1.4)	2.7 (0.6)	3.3 (0.9)	3.1 (0.8)	3.5 (0.6)	13.4 (1.2)	9.3 (1.0)	7.2 (0.8)
Non-Hispanic Black	41.3 (2.0)	**25.9 (1.8)**	**5.8 (1.2)**	**2.1 (0.6)**	**0.8 (0.3)**	3.7 (0.8)	**2.6 (0.4)**	**1.4 (0.4)**	6.8 (0.9)
Hispanic	40.2 (4.5)	20.8 (3.6)	**4.8 (1.4)**	2.3 (1.0)	1.9 (0.9)	5.1 (1.8)	11.5 (3.1)	11.1 (3.3)	7.9 (2.3)
Non-Hispanic Other Group^b^	39.4 (2.9)	25.3 (2.3)	**4.4 (1.2)**	2.5 (0.7)	**1.7 (0.5)**	4.2 (1.1)	**6.1 (1.2)**	**3.1 (0.8)**	7.6 (1.4)
Education									
High school diploma or less	**45.5 (1.8)**	**28.4 (1.8)**	4.9 (1.1)	2.2 (0.6)	1.5 (0.5)	4.3 (0.9)	**5.7 (0.9)**	**4.4 (0.8)**	**5.6 (0.9)**
Some college	**40.0 (2.4)**	**23.2 (1.9)**	3.4 (0.8)	2.7 (0.8)	**2.2 (0.7)**	**5.0 (1.0)**	**6.2 (0.9)**	**3.2 (0.6)**	8.4 (1.1)
Bachelor’s degree or more (ref)	31.5 (2.4)	15.5 (1.7)	4.2 (1.0)	2.9 (0.8)	1.4 (0.4)	2.7 (0.7)	10.8 (1.6)	7.8 (1.5)	8.7 (1.2)
Household income									
< $25,000	**43.3 (2.1)**	**25.5 (1.9)**	4.9 (1.1)	2.5 (0.8)	2.0 (0.5)	4.4 (1.1)	**5.8 (1.0)**	**3.7 (0.8)**	6.5 (1.1)
$25,000–$50,000	41.1 (2.3)	25.2 (2.0)	3.1 (0.8)	2.8 (0.8)	2.0 (0.6)	3.7 (1.0)	6.8 (1.0)	4.7 (0.8)	8.3 (1.3)
> $50,000 (ref)	37.9 (2.6)	21.5 (2.0)	4.3 (1.0)	2.6 (0.7)	1.6 (0.5)	3.1 (0.8)	8.7 (1.5)	6.6 (1.4)	8.8 (1.2)
Body Mass Index									
< 25 (ref)	37.3 (2.5)	21.2 (1.9)	4.4 (1.0)	2.1 (0.6)	2.0 (0.6)	3.7 (0.9)	6.0 (1.1)	3.9 (0.9)	7.8 (1.0)
25–29.9	41.5 (2.2)	**25.6 (2.0)**	3.9 (0.8)	2.3 (0.6)	1.4 (0.4)	3.1 (0.7)	7.6 (1.2)	4.6 (0.9)	6.8 (0.9)
> =30	41.5 (2.0)	23.4 (1.7)	4.5 (1.1)	3.1 (1.0)	1.9 (0.6)	4.6 (0.9)	7.4 (1.1)	5.1 (0.9)	7.6 (1.3)
Shopping frequency									
At least daily	**44.4 (2.3)**	**26.3 (2.0)**	4.5 (1.1)	2.6 (0.9)	2.1 (0.6)	3.0 (0.8)	6.3 (1.0)	4.4 (0.9)	**5.4 (0.9)**
1–6 times per week	39.8 (2.0)	23.3 (1.8)	3.6 (1.0)	2.5 (0.7)	1.7 (0.5)	4.5 (0.9)	8.4 (1.2)	**5.5 (1.0)**	**6.8 (1.0)**
Less than 1 time per week (ref)	36.3 (2.4)	20.3 (1.9)	3.2 (0.8)	2.5 (0.7)	1.3 (0.5)	4.6 (1.1)	6.6 (1.1)	3.8 (0.8)	10.3 (1.3)
Neighborhood Poverty Status^c^									
High Poverty	43.7 (2.4)	**26.0 (2.1)**	5.1 (1.4)	2.5 (0.8)	1.8 (0.5)	4.2 (1.2)	6.4 (1.3)	**3.3 (1.0)**	5.3 (1.1)
Less Poverty (ref)	39.2 (2.1)	21.7 (1.7)	4.1 (0.9)	2.6 (0.7)	1.8 (0.5)	4.4 (0.9)	7.8 (1.0)	5.7 (0.8)	7.5 (0.9)
Neighborhood Food Desert Status^d^									
Low-income/ Low-access	41.7 (2.5)	23.0 (2.1)	5.0 (1.1)	2.7 (0.9)	1.7 (0.4)	4.0 (0.9)	6.0 (1.0)	**3.3 (0.8)**	7.3 (1.3)
Not low-income/ Not low-access (ref)	39.8 (2.0)	22.7 (1.7)	4.2 (0.9)	2.5 (0.6)	2.2 (0.8)	4.5 (0.9)	8.0 (1.0)	5.7 (0.9)	6.8 (0.8)

All models controlled for customer age, sex, race/ethnicity, a city effect (study design), time trends, and included store identification as a random effect due to nesting of customers within stores. Boldface indicates statistically significant difference from reference category(*P* ≤ 0.05). *ASB *artificially-sweetened beverages; ref., reference category; *SSBs* sugar-sweetened beverages

^a^ Unsweetened water

^b^ Non-Hispanic Other Group included: *n* = 9 Non-Hispanic Pacific Islander/Native Hawaiian, *n* = 99 Non-Hispanic Asian, *n* = 123 Non-Hispanic American Indian/ Native Alaskan, *n* = 128 Non-Hispanic More than 1 racial group, *n* = 132 Non-Hispanic Racial Category not captured

^c^ *n* = 2741 customers with residential neighborhood data. High poverty status, > 50% of residents < 185% federal poverty level; Less poverty status, **≤** 50% of residents < 185% federal poverty level

^d^ *n* = 2741 customers with residential neighborhood data. Food desert neighborhoods were defined as census tracts that were both low-access (100 or more households without access to a vehicle and located > ½ mile from nearest supermarket) and low-income (either a poverty rate > 20% or the census tract’s median family income is < 80% of the state or metropolitan area’s median family income [41]

Across educational attainment, customers with a high school diploma or less, compared to those with bachelor’s degree or higher, had significantly greater purchasing of overall SSBs (45.8% vs. 31.5%; *P* < .0001) and soda while significantly less purchasing of overall ASBs (5.7% vs. 10.8%; *P =* 0.0001), diet soda, and unsweetened water (5.6% vs. 8.7%; *P =* 0 .02). Compared to customers with at least a bachelor’s degree, those with some college education also had greater overall SSB (40.0% vs. 31.5%; *P =* 0.0002), soda, energy drink, and sweetened tea purchasing and less overall ASB (6.2% vs. 10.8%; *P* < .0001) and diet soda purchasing. Largely similar socio-economic patterns of greater SSB and less ASB purchasing were observed across annual household income levels as well as the poverty status and food desert status of customers’ residential neighborhoods; though, differences were small and most not statistically significant.

Across shopping frequency, customers that shopped daily at the store had significantly greater overall SSB (44.4% vs. 36.3%; *P* = 0.002) and soda purchasing compared to customers shopping there less than once per week. Diet soda purchases were significantly more common among weekly shoppers compared those who shopped there less than once per week. Unsweetened water purchases were significantly less among both daily (5.4%; *P* = 0.008) and weekly (6.8%; *P* = 0.01) shoppers compared customers that shop less than once per week (10.3%).

## Discussion

Using direct observation data from a repeated cross-sectional sample of adult food and beverage customers, we examined SSB, ASB, and unsweetened water purchases at urban convenience and other small food stores over time (2014–2017) and across customer characteristics. The prevalence of both SSB and ASB purchases did not change across time, however, there were significant increases in the purchases of unsweetened water. Several differences in beverage purchases were identified across customer characteristics, which reflects different customer groups targeted to purchase SSBs and ASBs. Overall, findings demonstrate that significant work is needed to curb sweetened beverage and promote unsweetened water purchasing at convenience and other small food stores.

Despite promising trends in reduced SSB consumption and purchasing in the US [15, 17, 18, 21, 22, 24] since its peak in 2000 [19], we identified that nearly 1 in 2 food/beverage purchasers at convenience and other small food stores included an SSB and this high proportion of SSB purchasing did not significantly decrease from 2014 to 2017. Some of the disparity of our results with prior research may relate to our cohort of understudied convenience retail outlets capturing “on-the-go” purchasing, being limited to a small US geographic area, and/or study years—as most prior investigations ended before or near the start of our time frame (2014). A few recent trends bolster the flattened SSB purchase trend we identified. Jiang et al. (2020) identified a similarly flat 2014–2017 pattern in SSB consumption among adults in New York City [45], and industry sales of sugary drink calories per person per day have demonstrated a plateaued trend since 2013 [19, 46].

While we also identified stable trends in ASB purchases, water purchases increased significantly. Similar patterns of water have been identified by other studies using dietary intake [17, 20] and point-of-sales volume data at pharmacies [21]. However, ready-to-drink water was the least commonly purchased beverage type in our sample. Without complimentary evidence of decreases in SSB and ASB purchasing, these results suggest few, if any, customers were swapping water for their sweetened beverage selection.

We also identified SSB and ASB purchasing varied by key social groups that may be targeted differently by the beverage industry [33–35]. Aligning with prior SSB intake studies [15, 17, 19, 47], we found SSB purchasing to be highest among men, young adults, and customers with lower education and economic resources. In comparison, ASB and diet soda purchasing was highest among women, those 40–59 years, non-Hispanic White, Hispanic, and customers with higher education and economic resources. Given the robust evidence about the links between SSBs and poor health [2–7] and the increasing evidence for the harmful health effects of ASBs [4, 5, 8, 9], these results suggest that multiple population groups may all be at-risk for poor health outcomes due to their beverage choices. If future research continues to identify evidence of the harmful health impacts of ASBs, then a shift in public health may be required to focus on reducing all sweetened beverages (sugar- and artificially-) [14] and recognizing the greater diversity of social groups that may be affected.

Differing from prior research on SSB intake [17, 47], we did not identify differences in overall SSB purchasing by race/ethnicity at convenience and other small food stores. However, there were distinct purchasing patterns across SSB sub-types (e.g., higher purchasing of energy drinks among non-Hispanic White compared to non-Hispanic Black) as well as by overall ASB and diet soda purchasing. Such variation reflects the beverage industry’s product targeting of different sweetened beverages to specific racial/ethnic groups and highlights the need for additional research to investigate the ways diverse social groups may be exposed to different industry marketing messages and practices [34, 35].

Unsweetened water purchases showed fewer differences across customer characteristics compared to overall SSB and ASB purchases. Purchases were highest among customers with higher educational attainment and those that shopped less than once per week at the store. Shopping frequency was also associated with SSB purchasing with higher purchasing among daily compared to less frequent shoppers. Given the healthfulness of beverage selections varied by shopping frequency, the relative exposure to in-store marketing features at convenience and other small food stores may be an important contributor to the healthfulness of beverages selected by consumers [48–50] and deserves additional investigation.

### Strengths and limitations

These findings provide estimates for sweetened beverage and unsweetened water purchasing at venues underrepresented in previous research due to their known status of being highly challenging research venues. Using observational purchase data from convenience and other small food stores randomly-sampled and repeatedly measured across time, we analyzed recent trends and patterns in beverage purchases from 2014 to 2017. Despite these strengths, this study had several limitations. These data are limited to the specific region of Minneapolis-St. Paul, USA, and the narrow time frame (2014–2017) restricted our ability to assess longer trends over time. In addition, the process for determining customers’ age eligibility and the overall limited participation rate among customers may have introduced bias. Categories of customer characteristics were at times broad preventing comparisons among more refined groups (e.g., comparing customers with only a high school diploma to those who had not obtained a high school diploma). No explicit control for multiple models was employed, but we focused interpretation on the robust patterns observed in the associations with customer characteristics. We also focused on the proportion of customers making a beverage purchase and did not use other measures previously used to examine population patterns in beverage purchasing (e.g., volume sales, kilocalories purchased per person per day).

## Conclusion

While US research indicates improving trends in SSB and water purchasing over the past two decades, we found limited support for promising sweetened beverage trends at urban convenience and other small food stores from 2014 to 2017. Coupling this with industry evidence that sweetened beverages are an ever-growing share of revenue at convenience stores [32], suggests that interventions and policies specifically directed at US convenience and related outlets are a necessary focus to the work accomplished to date [14]. Fortunately, a recent policy evaluation [13] suggests excise taxes may be one effective tool in reducing purchases at these unique sites. At the same time, with many low-income communities relying on these outlets for their food and beverage needs [29, 51] and our results showing distinct patterns by socio-economic resources, it remains important to understand beverage purchasing as both a health and equity issue. Addressing the historical and social conditions that place marginalized communities at increased risk for SSB purchasing and limited water selections, including those at convenience stores, will be essential to improve population health as well as health equity.

## Data Availability

The datasets used and/or analysed during the current study are available from the corresponding author on reasonable request.

## Abbreviations

ACS: American Community Survey
ASB: artificially-sweetened beverage
BMI: body mass index
FPL: federal poverty level
SSB: sugar-sweetened beverage
STORE: STaple foods ORdinance Evaluation study
USDA: United States Department of Agriculture

## References

[1] Popkin BM, Hawkes C (2016). Sweetening of the global diet, particularly beverages: patterns, trends, and policy responses. Lancet Diabetes Endocrinol.

[2] Te Morenga L, Mallard S, Mann J. Dietary sugars and body weight: systematic review and meta-analyses of randomised controlled trials and cohort studies. BMJ. 2012;346:e7492. 10.1136/bmj.e7492 23321486

[3] Te Morenga LA, Howatson AJ, Jones RM, Mann J (2014). Dietary sugars and cardiometabolic risk: systematic review and meta-analyses of randomized controlled trials of the effects on blood pressure and lipids. Am J Clin Nutr.

[4] Imamura F, O'Connor L, Ye Z, Mursu J, Hayashino Y, Bhupathiraju SN (2016). Consumption of sugar sweetened beverages, artificially sweetened beverages, and fruit juice and incidence of type 2 diabetes: systematic review, meta-analysis, and estimation of population attributable fraction. Br J Sports Med.

[5] Malik VS, Li Y, Pan A, Koning LD, Schernhammer E, Willett WC (2019). Long-Term Consumption of Sugar-Sweetened and Artificially Sweetened Beverages and Risk of Mortality in US. Adults.

[6] Malik VS, Popkin BM, Bray GA, Després JP, Willett WC, Hu FB (2010). Sugar-sweetened beverages and risk of metabolic syndrome and type 2 diabetes: a meta-analysis. Diabetes Care.

[7] Singh GM, Micha R, Khatibzadeh S, Lim S, Ezzati M, Mozaffarian D (2015). Estimated global, regional, and National Disease Burdens Related to sugar-sweetened beverage consumption in 2010. Circulation..

[8] Pase MP, Himali JJ, Beiser AS, Aparicio HJ, Satizabal CL, Vasan RS (2017). Sugar- and artificially sweetened beverages and the risks of incident stroke and dementia: a prospective cohort study. Stroke..

[9] Chazelas E, Debras C, Srour B, Fezeu LK, Julia C, Hercberg S (2020). Sugary drinks, artificially-sweetened beverages, and cardiovascular disease in the NutriNet-Santé cohort. J Am Coll Cardiol.

[10] Chaloupka FJ, Powell LM, Warner KE (2019). The use of excise taxes to reduce tobacco. Alcohol Sugary Beverage Consumption.

[11] Farley TA, Halper HS, Carlin AM, Emmerson KM, Foster KN, Fertig AR (2017). Mass media campaign to reduce consumption of sugar-sweetened beverages in a rural area of the United States. Am J Public Health.

[12] Sugar-sweetened beverage taxation in the Region of the Americas. Washington, D.C: Pan American Health Organization; 2020.

[13] Bleich SN, Lawman HG, LeVasseur MT, Yan J, Mitra N, Lowery CM (2020). The association of a sweetened beverage tax with changes in beverage prices and purchases at independent stores. Health Aff.

[14] Krieger J, Bleich SN, Scarmo S, Ng SW. Sugar-Sweetened Beverage Reduction Policies: Progress and Promise. 2021;42(1):439–61. 10.1146/annurev-publhealth-090419-103005 33256536

[15] Vercammen KA, Moran AJ, Soto MJ, Kennedy-Shaffer L, Bleich SN (2020). Decreasing Trends in Heavy Sugar-Sweetened Beverage Consumption in the United States, 2003 to 2016. J Acad Nutri Dietetics..

[16] Mesirow MSC, Welsh JA (2015). Changing Beverage Consumption Patterns Have Resulted in Fewer Liquid Calories in the Diets of US Children: National Health and Nutrition Examination Survey 2001–2010. J Acad Nutri Dietetics..

[17] Bleich SN, Vercammen KA, Koma JW, Li Z. Trends in Beverage Consumption Among Children and Adults, 2003–2014. 2018;26(2):432–41. 10.1002/oby.22056 29134763

[18] Kit BK, Fakhouri TH, Park S, Nielsen SJ, Ogden CL (2013). Trends in sugar-sweetened beverage consumption among youth and adults in the United States: 1999-2010. Am J Clin Nutr.

[19] America HF (2018). Sugary drinks in America: Who's drinking what and how much?.

[20] Vieux F, Maillot M, Rehm CD, Barrios P, Drewnowski A (2020). Trends in tap and bottled water consumption among children and adults in the United States: analyses of NHANES 2011–16 data. Nutr J.

[21] Rummo PE, Pho N, Bragg MA, Roberto CA, Elbel B. Trends in Store-Level Sales of Sugary Beverages and Water in the U.S., 2006–2015. Am J Prev Med 2020;59(4):522–529. 10.1016/j.amepre.2020.04.022 32951682

[22] Piernas C, Ng SW, Popkin B. Trends in purchases and intake of foods and beverages containing caloric and low-calorie sweeteners over the last decade in the United States 2013;8(4):294–306. 10.1111/j.2047-6310.2013.00153.x PMC3711951 23529974

[23] Ng SW, Slining MM, Popkin BM (2014). Turning point for US diets? Recessionary effects or behavioral shifts in foods purchased and consumed. Am J Clin Nutr.

[24] Valizadeh P, Popkin BM, Ng SW (2020). Distributional changes in U.S. sugar-sweetened beverage purchases, 2002-2014. Am J Prev Med.

[25] Dunford EK, Miles DR, Ng SW, Popkin B (2020). Types and Amounts of Nonnutritive Sweeteners Purchased by US Households: A Comparison of 2002 and 2018 Nielsen Homescan Purchases. J Acad Nutri Dietetics..

[26] Kiszko K, Cantor J, Abrams C, Ruddock C, Moltzen K, Devia C (2015). Corner store purchases in a low-income Urban Community in NYC. J Community Health.

[27] Lent MR, Vander Veur S, Mallya G, McCoy TA, Sanders TA, Colby L (2015). Corner store purchases made by adults, adolescents and children: items, nutritional characteristics and amount spent. Public Health Nutr.

[28] Ruff RR, Akhund A, Adjoian T (2016). Small convenience stores and the local food environment: an analysis of resident shopping behavior using multilevel modeling. Am J Health Promotion.

[29] Madsen KA, Falbe J, Olgin G, Ibarra-Castro A, Rojas N (2019). Purchasing patterns in low-income neighbourhoods: implications for studying sugar-sweetened beverage taxes. Public Health Nutr.

[30] Gittelsohn J, Laska MN, Karpyn A, Klingler K, Ayala GX (2014). Lessons learned from small store programs to increase healthy food access. Am J Health Behav.

[31] Einav L, Leibtag ES, Nevo A. On the accuracy of Nielsen Homescan data. United States Department of Agriculture Economic Research Report Number 69 2008. Report No.: No. 1477–2016-121081.

[32] Crompton T. Convenience Stores in the US. IBISWorld; February 2021. Report No.: US INDUSTRY (NAICS) REPORT 44512.

[33] Nguyen KH, Glantz SA, Palmer CN, Schmidt LA. Transferring Racial/Ethnic Marketing Strategies From Tobacco to Food Corporations: Philip Morris and Kraft General Foods 2020;110(3):329–336. 10.2105/AJPH.2019.305482 PMC7002936 31944842

[34] Grier SA, Kumanyika S (2010). Targeted marketing and public health. Annu Rev Public Health.

[35] Lucan SC, Maroko AR, Sanon OC, Schechter CB (2017). Unhealthful food-and-beverage advertising in Subway stations: targeted marketing, vulnerable groups, dietary intake, and poor health. J Urban Health.

[36] Laska MN, Caspi CE, Lenk K, Moe SG, Pelletier JE, Harnack LJ, et al. Evaluation of the first U.S. staple foods ordinance: impact on nutritional quality of food store offerings, customer purchases and home food environments. Int J Behav Nutri Physical Activity. 2019;16(1):83. 10.1186/s12966-019-0818-1 PMC6751624 31533737

[37] Winkler MR, Lenk KM, Erickson DJ, Caspi CE, Laska MN. Longitudinal Fruit and Vegetable Sales in Small Food Retailers: Response to a Novel Local Food Policy and Variation by Neighborhood Socioeconomic Status 2020;17(15):5480. 10.3390/ijerph17155480 PMC7432731 32751326

[38] Pelletier JE, Caspi CE, Schreiber LRN, Erickson DJ, Harnack L, Laska MN (2016). Successful customer intercept interview recruitment outside small and midsize urban food retailers. BMC Public Health.

[39] D F, BH S, K C, IM B. Computerized collection and analysis of dietary intake information. Comput Methods Prog Biomed 1989;30(1):47–57. 10.1016/0169-2607(89)90122-3 2582746

[40] Bureau USC. American community survey (ACS) [cited 2020 February 20]. Available from: https://www.census.gov/programs-surveys/acs.

[41] Agriculture USDo. ERS food food access research atlas documentation [cited 2021 April 2]. Available from: https://www.ers.usda.gov/data-products/food-environment-atlas/documentation/.

[42] Caspi CE, Winkler MR, Lenk KM, Harnack LJ, Erickson DJ, Laska MN (2020). Store and neighborhood differences in retailer compliance with a local staple foods ordinance. BMC Public Health.

[43] (ASPE) OOTASFPAE. PRIOR HHS POVERTY GUIDELINES AND FEDERAL REGISTER REFERENCES: U.S. Department of Health and Human Services; [cited 2021 April 2]. Available from: https://aspe.hhs.gov/prior-hhs-poverty-guidelines-and-federal-register-references.

[44] Health MDo. reTHINK your drink! Every sip counts.: Minneapolis Health Department; n.d. [cited 2021 May 20]. Available from: https://rethinkyourdrink.minneapolismn.gov/.

[45] Jiang N, Yi SS, Russo R, Bu DD, Zhang D, Ferket B (2020). Trends and sociodemographic disparities in sugary drink consumption among adults in new York City, 2009–2017. Prev Med Rep.

[46] Economics KPP. 2025 Beverage calories initiative: report on 2019 progress toward the National Calorie Goal. Washington, DC.: Keybridge; 2020.

[47] Rosinger A, Herrick K, Gahche J, Park S. Sugar-sweetened Beverage Consumption Among U.S. Adults, 2011–2014. NCHS Data Brief. 2017(270):1–8. 28135185

[48] Cohen DA, Collins R, Hunter G, Ghosh-Dastidar B, Dubowitz T (2015). Store impulse marketing strategies and body mass index. Am J Public Health.

[49] Hecht AA, Perez CL, Polascek M, Thorndike AN, Franckle RL, Moran AJ. Influence of Food and Beverage Companies on Retailer Marketing Strategies and Consumer Behavior. Int J Environ Res Public Health. 2020;17(20):7381. 10.3390/ijerph17207381 PMC7600709 33050424

[50] Cohen DA, Bogart L, Castro G, Rossi AD, Williamson S, Han B (2018). Beverage marketing in retail outlets and the balance calories initiative. Prev Med.

[51] Gustafson A (2017). Shopping pattern and food purchase differences among supplemental nutrition assistance program (SNAP) households and non-supplemental nutrition assistance program households in the United States. Prev Med Rep.

